# Communication and information-giving in high-risk breast cancer consultations: influence on patient outcomes

**DOI:** 10.1038/sj.bjc.6601502

**Published:** 2004-01-20

**Authors:** E A Lobb, P N Butow, A Barratt, B Meiser, C Gaff, M A Young, E Haan, G Suthers, M Gattas, K Tucker

**Affiliations:** 1 Medical Psychology Research Unit, Department of Psychological Medicine, The University of Sydney, Sydney NSW 2006, Australia; 2 Hereditary Cancer Clinic, Prince of Wales Hospital, Sydney NSW 2031, Australia; 3 Screening and Test Evaluation Program, School of Public Health, The University of Sydney, Sydney NSW 2006, Australia; 4 Prince of Wales Hospital Clinical School, University of New South Wales, NSW 2052, Australia; 5 Genetic Health Services Victoria, Royal Children's Hospital, Parkville 3052, Australia; 6 Royal Melbourne Hospital, Parkville 3052, Australia; 7 Peter Mac Callum Cancer Institute, Melbourne, Victoria 3000, Australia; 8 South Australian Clinical Genetics Service, Women's and Children's Hospital, North Adelaide SA 5006, Australia; 9 Queensland Clinical Genetics Service, Royal Children's Hospital, Herston, Queensland 4006, Australia

**Keywords:** genetic counselling, familial breast cancer, outcomes of consultants’ communication

## Abstract

This longitudinal study aimed to document (i) the information-giving and patient-communication styles of *clinical geneticists and genetic counsellors (consultants)* in familial breast cancer clinics and (ii) assess the effect of these styles on women's knowledge, whether their expectations were met, satisfaction, risk perception and psychological status. A total of 158 women from high-risk breast cancer families completed self-report questionnaires at 2 weeks preconsultation and 4 weeks postconsultation. The consultations were audiotaped, transcribed and coded. Multivariate logistic regressions showed that discussing prophylactic mastectomy (*P*=0.00) and oophorectomy (*P*=0.01) led to women having significantly more expectations met; discussing genetic testing significantly decreased anxiety (*P*=0.03) and facilitating understanding significantly decreased depression (*P*=0.05). Receiving a summary letter of the consultation significantly lowered anxiety (*P*=0.01) and significantly increased the accuracy of perceived risk (*P*=0.02). Women whose consultant used more supportive communications experienced significantly more anxiety about breast cancer at the 4 weeks follow-up (*P*=0.00). These women were not significantly more anxious before genetic counselling. In conclusion, this study found that consultants vary in the amount of information they give and the way they communicate; and this variation can result in better or worse psychosocial outcomes. Greater use of supportive and counselling communications appeared to increase anxiety about breast cancer. Identifying methods to assist consultants to address emotional issues effectively may be helpful.

Women who are concerned about their cancer risk because of their family history should have access to accurate information and referral for cancer genetic counselling (including risk estimation). While many studies have explored women's expectations of the session, their risk perceptions, their psychological state and their testing and screening intentions, there is little information on what happens in a genetic counselling session and how this process affects patient outcomes.

Outcome studies in genetic counselling have largely relied on women's subjective assessment of the content and process of counselling, with few including an objective measure of what actually happened during the consultation. Furthermore, outcomes are often restricted to measures of satisfaction and understanding of risk. Such studies create a rather simplistic view of the goals of genetic counselling (Emery *et al*, 1972; Kessler, 1981).

Furthermore, many of these studies did not evaluate the role of patient's sociodemographic factors (Evers Kiebooms and Van den Berghe, 1979; Marteau and Richards, 1996). The samples comprised women being counselled for a wide array of conditions, including chromosomal, autosomal recessive and a small proportion of autosomal dominant conditions, did not adequately control for or measure counsellor variability and used *ad hoc* measures of patient's input and outcome variables (Michie, 1993).

The current study describes the process of genetic counselling for women from familial breast cancer families as shown by the analysis of audiotapes of counselling consultations. It examines the effect of different consultant communication styles on a variety of outcomes.

It was hypothesised that the more the consultant facilitated communication and the more information was provided, the better would be patient outcomes; (a) higher knowledge; (b) more accurate risk perception; (c) higher total satisfaction, satisfaction with the information given and satisfaction that expectations were met; (d) more expectations being met; (e) lower generalised anxiety and depression; and (f) lower anxiety about breast cancer.

## METHODS

### Participants

Women from high-risk breast cancer families who were attending their first consultation before genetic testing, in any one of 10 familial cancer clinics in four Australian States, were included in the study. Four clinical geneticists, one oncologist and two genetic counsellors conducted consultations. Women were quota-sampled according to whether or not they had previously had breast cancer. Women were considered ineligible for participation if they were unable to give informed consent, that is, if they were younger than 18 years or showed evidence of a severe mental illness. Individuals with limited literacy in English were also excluded because data collection was based on a self-administered questionnaire. None of the women had had a predictive test for, or was an obligate carrier of, a BRCA1 or BRCA2 mutation.

Of the 231 women who met the eligibility criteria, 27 women did not attend their appointment and 11 declined participation. Of the remaining 193 women, 158 women completed baseline (and follow-up) questionnaires, for whom there was an audible audiotape of their consultation.

A sample size of 158 women will detect a difference of 1.3 scores on the Hospital Anxiety and Depression Scale anxiety subscale as a result of consultants’ communication with 82% power at a significance level of *P*=0.05. This difference corresponds to an effect size of 0.32 (i.e. a small to medium effect), according to Cohen's (1988) definition.

### Procedure

This study is one component of a larger randomised controlled trial of providing women with an audiotape of their genetic counselling consultation (Lobb *et al*, 2002a), and the influence of patient characteristics on consultants’ communication (Lobb *et al*, 2002b). Staff at each of the participating clinics invited women to participate in the study (between November 1998 and May 2000) when they telephoned to make their appointment. If verbal agreement was obtained, women were mailed self-administered questionnaires 2 weeks before and 4 weeks after their genetic consultation. The consultations were audiotaped and copies of the audiotape were retained for analysis. Ethics approval was obtained from 10 different ethics committees prior to data collection.

### Coding of transcripts of audiotapes

A detailed coding system and manual for the transcribed audiotapes were devised. The transcripts were coded to capture 10 aspects of genetic counselling including (a) *information giving concerning*: (i) breast cancer genetics, (ii) genetic testing, (iii) family history and risk, (iv) prophylactic surgery, (v) breast cancer prevention, (vi) screening and management; (b) *Communication style*: including (vii) facilitating patient involvement, (viii) facilitating understanding, (ix) patient centredness and partnership building and (x) supportive and counselling communications. These categories were based on (a) the National Health & Medical Research Council's Guidelines on the Familial Aspects of Cancer: A Guide to Clinical Practice (NH&MRC, 1999) and (b) an Australian survey of clinical geneticists/genetic counsellors describing their practice (Lobb *et al*, 2001) and (c) studies that identified women's expectations of the genetic counselling session (Julian-Reynier *et al*, 1996, 1998; Hallowell *et al*, 1997; Michie *et al*, 1997; Veach *et al*, 1999; Brain *et al*, 2000).

Under each of these categories, the content or types of communication that characterised that issue were identified. The presence or absence of each component was coded. Whether the woman or the consultant initiated the content was noted and, finally, the actual words used were recorded. An example of coding for an information variable is shown in Table 1,

**Table 1:** Example of Information-giving variables

**Content of category**	**% of consultations in which it occurred**
*Screening and management recommendations*	
Breast Cancer Screening	
Mammography	89
Risks	19
Benefits	18
Direct recommendation	68
Screening other female family members	42
Ultrasound	34
Clinical breast examination	67
Breast self-examination	62
Tamoxifen	58
Risks	32
Benefits	27
Tamoxifen clinical trial	41
Ovarian cancer screening	
Pelvic ultrasound	40
CA125 blood test	24
Risks	32
Benefits	27

and a communication style variable is shown in Table 2

**Table 2:** Example of communication styles

*Supportive or counselling communication*	
Checking patient concerns	56%
Discussing feelings about coming to clinic	32%
Discussing feelings about own/family br.ca.	63%
Discussing feelings about having genetic test	36%
Discussing feelings of being at risk	44%
Discussing emotional concerns	57%
Discussing social concerns	22%
Discussing medical concerns (not genetics)	12%
Offering follow-up appointment	57%

. For example, the category of *Information on Screening and Management* had 16 component topics that were summed to provide a total score.

### Coding reliability

Three coders (including EL) were trained. Two coders recoded a random 10% of their own consultation transcripts and 10% of the other coder's transcripts. The average interrater reliability over the 274 codes was 93% (range 67–100%) and the average intrarater reliability was 92% (range 65–100%). The areas of highest agreement were information-giving categories (e.g. risk (96–100%); screening (89–99%); and there were lower levels of agreement on some consultant communication styles: facilitating communication (67–87%) and discussing psychosocial issues (69–76%). Thus, interpretation of findings on these latter variables must be viewed with some caution.

## MEASURES

### Predictors

#### Demographic characteristics

Women were asked to provide details on age, education, occupation, marital status, medical or allied health training, and the number of biological children and sex of each child.

### Outcomes

#### Breast cancer genetics knowledge

Before and after the consultation, an eight-item true–false measure derived from one developed by Lerman *et al* (1996) assessed knowledge about breast cancer genetics.

#### Expectations

Prior to the consultation, women indicated on a five-point scale ranging from ‘not at all important’ to ‘very important’ their response to seven possible reasons for attending a genetic clinic and similarly rated nine possible information topics that they might want covered at their first appointment. This scale was developed for the purposes of this study and included items suggested by expert opinion, the literature and structured telephone interviews with at-risk women. The total scores of expectations (possible range 0–7) and information sought (range 0–9) were calculated and compared to what women experienced according to the coded transcribed consultation.

#### Perceived risk

Before and after the consultation, women were asked to estimate their risk of developing breast cancer over their lifetime (or if affected, a second breast cancer) by choosing between nine response options ranging from 1 in 100 (1%) to inevitable (100%).

Participants’ numerical estimate of lifetime risk was converted to a category, according to the figures given in the Australian National Health & Medical Research Council Guidelines, for example, a potentially high-risk category (25–80% lifetime risk of breast cancer), a moderate-risk category (12–25%) and an average-risk category (9–12%) (NH&MRC, 1999). These figures were constructed acknowledging the limitations of risk analysis, based on data with very wide confidence intervals. Standard methods such as the Gail Model or Claus data are not commonly used in Australia, particularly in defining high risk. According to these NH&MRC guidelines, 60% of unaffected participants in this study were categorised as potentially high risk, 31% as moderate risk and 9% as at an average risk of developing breast cancer.

#### Objective risk

This was determined by the figure given by the consultant in the consultation or the postconsultation summary letter (all women received a figure in either of these communications). Participants’ responses were deemed *accurate* if their risk estimate fitted within the risk category given by the consultant. If a woman's perception of risk fell on a cutoff point of categories (12–25%), they were deemed accurate if either of the categories in which they could be placed corresponded with that given by the consultant. This method would tend to increase the percentage of those considered accurate compared to other methods. However, we felt that this was a more valid approach as it reflects the actual figures (or words) given in consultations. If women were inaccurate, it was determined whether they had underestimated or overestimated their risk of breast cancer.

Objective risk could be calculated only for unaffected women, as in the majority of consultations (61%) and follow-up letters of affected women, no risk figure or category was given of the chances of getting a second breast cancer.

#### Breast cancer anxiety

This was measured before and after the consultation using the Impact of Events Scale, a 15-item reliable and validated scale measuring intrusion and avoidance responses in relation to a specific stressor (Horowitz *et al*, 1979; Thewes *et al*, 2001). In the current study, the particular stressor was concern about being at risk of developing breast cancer for unaffected women and concern about developing a second cancer for affected women. Scores above 40 on either scale indicate a significant stress response.

#### General anxiety and depression

This was measured before and after the consultation by the 14-item Hospital Anxiety and Depression Scale, which is a valid and reliable measure. It consists of two subscales of seven items assessing anxiety and depression (Zigmond and Snaith, 1983). Questions have four response options, giving scores ranging from 0 to 21 for each subscale. A score of higher than 10 on either subscale is an indication of clinical anxiety or depression.

#### Satisfaction with the genetic counselling session

Satisfaction was measured after the consultation using a modified version of the 12-item short form of the 36-item ‘Satisfaction with Genetic Counselling Scale’, developed by Shiloh *et al* (1990). This shorter version of the scale is highly correlated with the full scale (*r*=0.90) and has good reliability (Cronbach *α*=0.78).

### Statistical methods

Descriptive statistics were used to summarise most of the data, including demographics and psychological status. Frequencies were calculated for individual counsellor communication styles. The total scores for the 10 predefined counselling categories were calculated by summing the component items.

Univariate analyses exploring associations between (a) demographic variables (education, occupation, medical/allied health training, age and number of daughters), psychological status, disease status (affected/unaffected) and consultation styles; and (b) the outcomes listed above were undertaken using parametric statistics (*t*-tests and Pearson's correlations) if the outcome score was normally distributed, and nonparametric statistics (Mann–Whitney *U* and Spearman's correlations) if the outcome total score was non-normally distributed. Some individual consultation communications were analysed by *χ*^2^ analyses. Variables associated with the outcomes at *P*<0.25 (Hosmer and Lemeshoe, 1989) were included in multivariate analyses These analyses explored the effect of consultant communications on outcomes, controlling for potential confounders.

## RESULTS

A total of 89 women were unaffected with breast cancer (56%) and 69 women were affected (44%). None were affected with ovarian cancer. The majority had a family history of breast cancer only (77%) and almost a quarter had a family history of breast and ovarian cancer (23%). The demographic characteristics of the women are shown in Table 3

**Table 3:** Demographic characteristics of sample (*n*=158)

	**Category**	**Unaffected (*n*=89)**	**Affected (*n*=69)**
Age		Mean 38.65 years (s.d. 9.0) (range 19–60)	51.36 (s.d. 11.5) (range 28–79)
Marital status	Married	73.9%	79.7%
	Not married	26.1%	20.3%
Educational level	Below HSC (year 12)	36.0%	56.3%
	Above HSC	64.0%	43.7%
Occupation	Professionals	59.3%	53.6%
	Nonprofessional	40.1%	46.4%
Allied health trained	Yes	35.6%	29.0%
	No	64.4%	71.0%
*Children*			
Girls	No girls	49.4%	24.6%
	1 or more	50.6%	75.4%
Boys	No boys	53.9%	39.1%
	1 or more	46.1%	60.9%
Risk status	High-risk	60%	
	Moderate	31%	
	Average	9%	

Not all categories sum to 100 due to missing data.

.

### Overall impact of consultations on patient outcomes

At baseline, the mean number of correct answers to knowledge items was 5.1. (s.d. 1.76) out of a possible 8. At the 4-week follow-up, the median increase in knowledge as a result of genetic counselling was 1.00 (s.d. 1.52, range −3 to 6).

The mean number of expectations met in the consultation was 4.06 (s.d. 1.70, range 0–7). Pearson's correlations showed that meeting women's expectations was unrelated to their overall satisfaction with the genetic counselling session (*r*=0.001, *P*=0.99), their satisfaction with the information they received (*r*=0.069, *P*=0.42) or satisfaction that their expectations were met (*r*=0.081, *P*=0.34).

The mean change in anxiety score was −0.4308 (s.d. 2.97, range −9 to 6) and that for depression −1.6923 (s.d. 2.56, range −9 to 7). The mean change score in anxiety about breast cancer was −0.9256 (s.d. 4.52, range −15 to 12).

### Risk perceptions at follow-up

At follow-up, 70% of unaffected women accurately estimated their risk (compared to 50% at baseline). In total, 20% of unaffected women underestimated their risk at follow-up (compared to 27% at baseline) and 10% overestimated it (compared to 23% at baseline).

### Satisfaction with the genetic counselling session

In total, 95% of women felt that the consultant had explained their situation clearly and 89% felt that their expectations were met. In all, 82% thought the consultant showed enough dedication, 86% felt that the consultant understood what was bothering them and 96% felt listened to. Finally, 84% were satisfied with the information they received. The two areas where women, both unaffected and affected, were less satisfied were in feeling reassured (69 and 68%, respectively) and that the consultation helped them cope better with their situation (68 and 57%, respectively).

#### Multivariate analyses of the effect of consultants’ information-giving and counselling communications on women's outcomes

Information delivery: breast cancer genetics, genetic testing, family history, breast cancer prevention, screening and management and prophylactic surgery

Women who had more aspects of genetic testing discussed had a significant decrease in anxiety (median HADS anxiety change score minus 1.000 *vs* 0.2333). (*t*=−2.22, *P*=0.03) compared to those who had not.

Women who had prophylactic mastectomy and oophorectomy discussed reported significantly more expectations met (mean 4.76 and 4.92, respectively), than women who did not have these discussed (mean 3.25 and 3.46, respectively) (OR=7.34, 95% CI=1.96–27.57, *P*=0.003) for mastectomy and (OR=17.72, 95% CI=2.07–151.62, *P*=0.009) for oophorectomy. There was also a trend for discussion of these issues to be associated with a greater reduction in anxiety about breast cancer, but these associations did not reach significance (*P*<0.1). No other associations were found between information-giving and patient outcomes.

#### Facilitating understanding, facilitating involvement, partnership building, patient centredness and supportive and counselling communications

Women whose consultants facilitated understanding more had a significantly greater decrease in depression (median HADS change in depression score −1.093 *vs* −1.988). (*t*=−1.959, *P*=0.052).

Women whose consultants used four or more supportive communications were significantly more anxious about breast cancer at 4 weeks follow-up (median IES Intrusion change score 0.1458 *vs* −1.844). (OR=1.66,95% CI=1.25–2.19, *P*=0.000). We examined whether the women who received more supportive and counselling communications were more anxious at baseline than those women who received fewer, but a significant association was not found (*z*=−1.58, *P*=0.11).

#### Receiving a written summary of the genetic counselling session

Copies of the summary letter sent to women after their consultation were obtained for all women participating in the study. However, only 50% of the women had received a copy of their letter by the follow-up assessment. Of these, almost all (92%) reported that they had read it.

Multivariate analyses showed that women who had received a letter summarising their consultation had lower anxiety (median score −1.304 *vs* 0.544), OR=0.38, 95% CI=0.177–0.806, *P*=0.012, a trend towards less anxiety about breast cancer (median score −0.058 *vs* −1.78), OR=0.50, 95% CI=0.221–1.112, *P*=0.089 and increased risk accuracy (79% were accurate *vs* 58%) OR=2.61, 95% CI=1.139–6.017, *P*=0.023 compared to those women who had not received a summary letter.

A summary of significant results from multiple logistic and linear regression analyses of outcomes of consultants’ communications is shown in Table 4

**Table 4:** Summary of significant results from multiple logistic and linear regression analyses of outcomes of consultants’ communication

**Outcome**	**OR**	**95% CI**	***P*-value**	
*Consultant discussed prophylactic mastectomy*				
Expectations met (mean 4.76 *vs* 3.25)	7.34	1.96–27.57	0.003	
*Consultant discussed prophylactic oophorectomy*				
Expectations met (mean 4.92 *vs* 3.46)	17.72	2.07–151.62	0.009	
*Consultant use of supportive or counselling communications*				
Change in breast cancer anxiety (median 0.1458 *vs* −1.844)	1.66	1.25–2.10	0.001	
*Summary letter of consultation (received and read)*				
Risk perception Accuracy (79% *vs* 58%)	2.61	1.14–6.02	0.023	
Change in general anxiety (median −1.304 *vs* 0.544)	0.38	0.177–0.81	0.012	
				
**Consultant discussed genetic testing**	***β***	**95%CI**	***t***	***P***
Change in general anxiety (median change score −1.000 *vs* 0.2333)	−0.204	−0.542–−0.031	−2.22	0.028
*Consultant facilitated understanding*				
Change in depression (median change score −1.093 *vs* −1.988)	−0.171	−0.479–0.002	−1.959	0.052

.

## DISCUSSION

This study has provided a detailed analysis of the process of genetic counselling in familial breast cancer within the Australian context. It has been the first study that has audiotaped, transcribed and coded in detail the process of genetic counselling in a large group of women who are affected and unaffected with breast cancer and who are members of potentially high-risk breast cancer families.

The role of the clinical geneticist and genetic counsellor in cancer genetic services is considerable. They routinely initiate contact with the family, obtain a pedigree, obtain consent from living relatives to access medical records, confirm relevant medical history, ascertain family beliefs about the inheritance pattern, advise family members of their risks and options and arrange clinical screening (Richards, 1993).

In recent reviews outlining the process of cancer genetic counselling (Kelly, 1999; Peters *et al*, 1999; Stopfer, 2000), the provision of emotional support and reassurance was identified as an important role for genetic counsellors. One author argues that genetic counselling must be psychodynamically oriented, recommending that the counsellor be sufficiently versed in psychology or psychiatry to recognise the emotions of the patient and the family members and to help them deal effectively with these emotions (Lynch and Lynch, 1996). This study explored the effect of communications targeting both understanding and emotional adjustment in genetic counselling consultations.

The high levels of breast cancer-related distress reported in other studies (Kash *et al*, 1992; Lerman *et al*, 1997; Watson *et al*, 1999) were not found in this study. However, around 18% of women were highly anxious and about 4% were significantly depressed. A surprising finding of this study was that the women who saw consultants who counselled and supported them were more anxious about breast cancer at follow-up. These women did not have elevated anxiety about breast cancer prior to their genetic consultation, so it does not appear that consultants were responding to existing high anxiety by providing more support, nor that these women had high anxiety that was resistant to change.

As long-term outcomes were not assessed in this study, the duration of heightened anxiety and its effect is not known. Increased anxiety is not necessarily inappropriate in the context of genetic counselling, as a family history of breast cancer can raise many emotional issues for women. For example, familial breast cancer can cause premature death and involve a number of female family members; hence loss and grief issues are frequently prominent for the woman. Perhaps both women and counsellors underestimate the impact of addressing these emotional concerns, possibly previously unacknowledged. Some authors suggest that women need to discuss their feelings of loss and anxiety about the future first, to enable them to focus on the genetic issues (Carter and Hailey, 1999; Stopfer, 2000). Therefore, we are not suggesting that the current finding be used as a rationale to reduce the counselling component of genetic counselling.

Australian providers of genetic counselling cited identifying the woman's individual needs and concerns as a major goal of genetic counselling (Lobb *et al*, 2001). Previous authors have argued that the success of the genetic counselling session is dependent upon the counsellor accurately gauging the woman's needs and expectations and that the woman's agenda be discussed at the beginning of the counselling session (Hallowell *et al*, 1997).

The consultant elicited the woman's agenda in 69% of consultations but contrary to our hypothesis, women who were asked their agenda did not have improved outcomes. Perhaps, consultants are not exploring women's agendas in sufficient detail, nor following through on information at this point. In another analysis of these data reported elsewhere, we found that consultants were not responsive to women's expectations or their level of psychological distress prior to the consultation (Lobb *et al*, 2002b).

This study found that women who had prophylactic mastectomy and oophorectomy discussed had significantly more expectations met. Hopwood (1997) has suggested that women attending genetic counselling clinics are likely to be information seekers who cope by monitoring their environment and taking action to reduce risk (Hopwood, 1997).

While the Australian NH&MRC National Best Practice Guidelines for Familial Cancer Clinics (1997) recommend that consultants discuss prophylactic mastectomy and oophorectomy with all women from high-risk families, perhaps some practitioners feel concerned about women's response to its discussion. Our findings suggest that women wish to discuss the option of prophylactic surgery and that discussing this area of management does not cause psychological distress.

While 77% of affected women indicated that they wanted to know their chances of developing a second cancer (in the contra lateral breast), this was not discussed in 61% of consultations, perhaps because consultants felt concerned that the woman was still adjusting to her current breast cancer diagnosis. Similarly, the majority of affected women (98%) wanted to know their family's risk of developing breast cancer and this was given in under half of the consultations (44%).

The provision of other information and also, surprisingly, facilitation of active involvement were not associated with patient outcomes. Perhaps, women in the current study became overloaded with information. The findings that providing a written summary after the consultation (a technique to simplify and summarise information) lessened anxiety and, importantly, increased their accuracy of risk perception after the session, supports previous research that found written summaries of genetic consultations to be beneficial (Hallowell and Murton, 1998). Importantly, the facilitation of understanding was associated with reduced depression at follow-up, suggesting that women struggle with the large amount of information provided and can feel worse if they are not helped to understand it.

### Future research and recommendations

Further research is needed to develop and evaluate interventions that identify and address anxiety levels in women attending for genetic counselling for familial breast and ovarian cancer. The increase in anxiety about breast cancer identified with increased supportive and counselling communications may be due to emotional issues being raised without adequate resolution. It may be helpful for consultants not only to assess psychological stress before the consultation but to also keep checking during the consultation as to how the woman is coping. Training programmes to assist clinicians identify and respond more consistently to the needs and concerns of women attending genetic counselling in the familial breast cancer setting may be helpful. Some consultants may consider such psychological strategies beyond their role and prefer a multidisciplinary approach where referral for appropriate counselling can be initiated.

Important findings of this study are that the provision of a summary letter after the consultation increased women's accuracy of risk perception and reduced anxiety, and that communications that facilitated understanding led to a greater reduction in depression. Few studies to date have been able to identify ways to improve women's risk perception after counselling. It is recommended that the practice of providing written summaries continue, that women are encouraged to review the letter and that other mechanisms to help women understand genetic information are explored. Finally, research into individual counsellor differences would be useful and investigation of more subtle communication behaviours than those measured here.

### Limitations of the study

It should be acknowledged that while verbal communication could be accurately coded from the audiotapes, it was not possible to capture nonverbal communication. It is possible that if nonverbal cues were included in the analysis quite a different result would have been obtained. Additionally, as has been identified in previous studies in the familial cancer setting, women in this study tended to be of higher education and professional status than the general population. The percentage of women with tertiary qualifications was 57% compared with 37% in the Australian population (Australian Bureau of Statistics, 1997). As there were few refusals to participate in the study (*n*=11), our study does seem to reflect accurately the population who attend for genetic counselling. Thus, while these findings may not be generalisable to less educated women, they do appear generalisable to the target population.
